# Mechanism of action and impact of thiol homeostasis on efficacy of an enzyme replacement therapy for classical homocystinuria

**DOI:** 10.1016/j.redox.2024.103383

**Published:** 2024-10-02

**Authors:** Thilo Magnus Philipp, Teodoro Bottiglieri, Wilmelenne Clapper, Kai Liu, Steve Rodems, Csaba Szabo, Tomas Majtan

**Affiliations:** a Department of Pharmacology, University of Fribourg, Faculty of Science and Medicine, Fribourg, Switzerland; b Center of Metabolomics, Institute of Metabolic Disease, Baylor Scott & White Research Institute, Dallas, TX, 75204, USA; c Travere Therapeutics, Inc., San Diego, CA, 92130, USA

**Keywords:** Cystathionine beta-synthase, Homocysteine, N-acetylcysteine, Enzyme replacement therapy, Homocystinuria, Disulfide isomerization, Plasma redox status

## Abstract

Homocystinuria (HCU) due to cystathionine beta-synthase (CBS) deficiency is characterized by elevated plasma and tissue homocysteine levels. There is no cure, but HCU is typically managed by methionine/protein restriction and vitamin B_6_ supplementation. Enzyme replacement therapy (ERT) based on human CBS has been developed and has shown significant efficacy correcting HCU phenotype in several mouse models by bringing plasma total homocysteine below the clinically relevant 100 μM threshold. As the reactive nature of homocysteine promotes disulfide formation and protein binding, and ERT is unable to normalize plasma total homocysteine levels, the mechanism of action of ERT in HCU remains to be further characterized. Here we showed that only a reduced homocysteine serves as a substrate for CBS and its availability restricts the homocysteine-degrading capacity of CBS. We also demonstrated that cells export homocysteine in its reduced form, which is efficiently metabolized by CBS in the culture medium. Availability of serine, a CBS co-substrate, was not a limiting factor in our cell-based model. Biological reductants, such as N-acetylcysteine, MESNA or cysteamine, increased the availability of the reduced homocysteine and thus promoted its subsequent CBS-based elimination. In a transgenic I278T mouse model of HCU, administration of biological reductants significantly increased the proportion of protein-unbound homocysteine in plasma, which improved the efficacy of the co-administered CBS-based ERT, as evidenced by significantly lower plasma total homocysteine levels. These results clarify the mechanism of action of CBS-based ERT and unveil novel pharmacological approaches to further increase its efficacy.

## Highlights

• Mechanism of Hcy degradation by CBS-based ERT in HCU is not fully understood.
• First, ERT degrades any free reduced Hcy available in plasma.
• Second, tissues export reduced Hcy, which is instantly degraded by ERT present in circulation.
• Third, ERT eliminates reduced Hcy released from dimers and plasma proteins.
• Alteration of plasma redox dynamics by the biological reductants could increase efficacy of the ERT.

## Graphical abstract

**Graphical abstract. Schematic representation of the proposed mechanism of action of the simultaneous application of CBS ERT and NAC**. (*Left*) Application of CBS45 and NAC in cell culture. CBS45 catalyzes the condensation of free, reduced Hcy with Ser or Cys to form cystathionine. This eliminates Hcy released from the cells during their growth and Hcy released from homo-/hetero-disulfides or protein conjugates by NAC. (*Right*) Application of CBS ERT and NAC in the plasma of HCU mice. Exogenous NAC, endogenous or dietary reducing agents can release Hcy from proteins and disulfides. This makes them available for renal clearance or catalysis by CBS ERT. In addition, CBS ERT eliminates the constant flow of endogenously produced and exported reduced Hcy from tissues.

**Figure:**
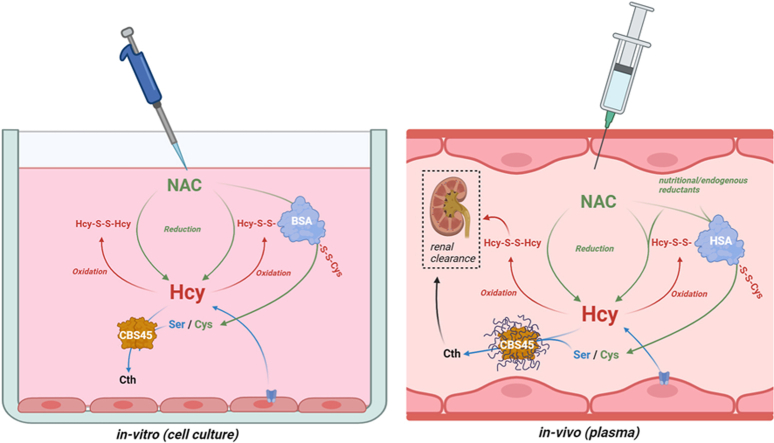

## 1 Introduction

Classical homocystinuria (HCU) is a rare inherited metabolic disorder characterized by the elevation of homocysteine (Hcy) levels in plasma and urine. It is the most common loss-of-function disease in the metabolism of sulfur-containing amino acids primarily caused by the pathogenic point mutations affecting cystathionine beta-synthase (CBS) [1]. CBS lies at the branch point where the fate of Hcy is decided: Hcy will either be remethylated back to methionine (Met) and thus will remain in the methionine cycle or be irreversibly committed to cysteine (Cys) synthesis via the transsulfuration pathway [2]. CBS, a unique enzyme with a multidomain architecture, relies on three cofactors: catalytically active pyridoxal-5′-phosphate (PLP), heme of an unclear function, and the allosteric activator S-adenosylmethionine (SAM) [3]. Missense mutations in the CBS gene result in a dysfunctional enzyme, which is unable to condense serine (Ser) and Hcy to cystathionine (Cth). Hence, metabolites upstream of the block accumulate, such as Hcy, Met, SAM and S-adenosylhomocysteine (SAH), while downstream metabolites including Cth and cysteine (Cys) are depleted.

The biochemical imbalance due to CBS deficiency translates into clinical symptoms of HCU, which encompass connective tissue defects leading to myopia, dislocated optic lenses, skeletal abnormalities and osteoporosis, cardiovascular events including thromboembolism and stroke, and cognitive impairment [1]. Although the pathophysiology of HCU is not yet fully understood, many of these symptoms can be attributed to the effects of massively elevated Hcy and its derivatives, such as Hcy thiolactone, in tissues and body fluids [4]. Importantly, the severity of the disease, as determined by the genotype and subsequent degree of CBS inactivation, varies widely. Early studies showed that the supplementation of vitamin B_6_ exerts therapeutic effects in some patients [5]. The phenomenon of “pyridoxine responsiveness” was clearly confirmed in a landmark natural history study [6]. This study was recently revisited and further stratified by correlating pyridoxine dose with plasma total Hcy (tHcy) levels and patient phenotype [7]. While pyridoxine responsive HCU patients can achieve normalization or good metabolic control solely with pharmacological doses of pyridoxine, pyridoxine non-responsive individuals are prescribed a low-protein (Met-restricted) diet often accompanied by Met-free amino acid formula to maintain positive nitrogen balance and betaine to promote alternative recycling of Hcy to Met [8]. Protein restriction is difficult to follow, time consuming and substantially decreases the quality of life of HCU patients, causing compliance issues, which often result in exacerbation of clinical symptoms [8,9].

To address the unmet therapeutic need within the HCU community, an enzyme replacement therapy (ERT) for HCU called pegtibatinase (previously known as 20NHS PEG-htCBS C15S, OT-58 or TVT-058) has been developed [10]. Pegtibatinase is a dimeric truncated human CBS lacking the C-terminal regulatory domain (Δ414-551) carrying C15S mutation to prevent disulfide bridge-based multimerization and chemically modified by 20 kDa linear polyethyleneglycol (PEG) chains to increase half-life and potentially prevent immunogenic reactions [11]. Pegtibatinase showed remarkable efficacy in preclinical studies. In several mouse models of HCU, pegtibatinase substantially decreased plasma and tissue Hcy levels, improved overall metabolic balance and rescued murine HCU phenotype, such as neonatal lethality of CBS knockout mice [12]. It also improved body composition, ameliorated facial alopecia and protected from the cognitive impairment in a transgenic HCU mouse model lacking mouse CBS, but expressing a pathogenic human CBS I278T transgene from zinc-inducible promoter (the transgenic I278T mouse model) [13,14]. All these beneficial effects of pegtibatinase in HCU mice were accomplished on a background of unrestricted Met intake. Combination of pegtibatinase administration with a mild protein restriction (about 50 % decrease in Met intake) resulted in full normalization of plasma tHcy levels, the effect which was not achieved even by a very severe Met restriction alone (about 90 % decrease in Met intake) [15]. The impressive preclinical efficacy along with a good safety profile [16] propelled pegtibatinase into evaluation in HCU patients advancing through the Phase 1/2 COMPOSE study (ClinicalTrials.gov ID# NCT03406611), and culminating in the currently on-going Phase 3 HARMONY study (ClinicalTrials.gov ID# NCT06247085) and Phase 3 ENSEMBLE long-term extension study (ClinicalTrials.gov ID# NCT06431893).

Despite the significant efficacy of pegtibatinase improving metabolic balance in HCU and correcting HCU-related phenotype by degrading accumulated Hcy in plasma [10,[12], [13], [14], [15]], the mechanistic details of pegtibatinase's action remain poorly understood. Pegtibatinase does not enter the cells and, upon absorption from a subcutaneous compartment, predominantly remains in the circulation [16]. Higher doses of pegtibatinase in repeated subcutaneous administration expectedly resulted in higher plasma levels of CBS activity in HCU mice, but doses over 8 mg/kg of body weight failed to further reduce plasma tHcy levels [16] suggesting that the plasma levels of pegtibatinase are not limiting factor for its efficacy. Only a combination of pegtibatinase with some degree of Met restriction resulted in a further decrease of plasma tHcy concentrations [15] suggesting that the flux of Hcy from tissues into bloodstream and availability of Hcy for enzymatic degradation represent important factors determining the efficacy of CBS-based enzyme therapy for HCU.

Here we present the results of a comprehensive investigation that elucidate the mechanism of Hcy-lowering action of CBS utilizing *in vitro* and *in vivo* approaches. Among others, we utilized a novel multimodal fluorescent probe for quantification of biological thiols and aimed to elucidate the mechanistic details of CBS-based ERT for HCU, with special attention to the underlying redox dynamics of biological thiols.

## 2 Materials and methods

**Chemicals and enzymes.** Unless stated otherwise, all chemicals were purchased from Sigma or Fisher Scientific. L-[U–14C]-Ser was obtained from PerkinElmer Life Sciences. Multimodal fluorescent Probe 1 specifically detecting Hcy, Cys and glutathione (GSH) was custom-synthesized by Enamine according to the chemical synthesis route published previously [17]. Truncated human (Δ414-551 also known as CBS45 and Δ2-39+Δ398-551 also known as CBS40) and yeast (Δ346-507 also known as tyCBS) CBS enzymes lacking the C-terminal regulatory domains and carrying permanent 6xHis-tag at their C-termini were recombinantly expressed in *E. coli* and purified using a two-step chromatographic process essentially as described previously [18]. PEGylation, a conjugation of the purified CBS with N-hydroxysuccinimide ester-activated polyethylene glycol (PEG) molecules of 5, 10 or 20 kDa in length (all from NOF), was carried out as reported previously [11]. The tyCBS modified with 20 kDa ME-200GS PEG (here further referred to as PEG-CBS) was used in the animal model, while CBS45 was used in the *in vitro* studies.

**Animals and study design.** A breeding pair of heterozygous transgenic C57BL6 mice knocked out for mouse CBS and expressing human pathogenic CBS I278T mutant transgene from zinc-inducible promoter was generously provided by Dr. Warren Kruger (Fox Chase Cancer Center, Philadelphia, PA, USA) [19]. Breeding pairs were maintained on water containing 25 mM zinc chloride to induce transgene expression to rescue the homozygous pups from neonatal death. After weaning at 21 days of age, mice were tagged, genotyped, switched to a regular water supply and maintained on extruded standard diet 2920X (Envigo, CA, USA) or 3436 (Kliba Nafag, Switzerland). Homozygous CBS knockout mice carrying the I278T transgene (further referred to as I278T mice) and their siblings with two copies of mouse CBS (further referred to as WT) were used for *in vivo* studies.

Plasma samples of mice used for a comparative evaluation of novel fluorescence-based quantification of biothiols were collected at the University of Fribourg under an animal protocol approved by the Food Safety and Veterinary Affairs Department of the Canton of Fribourg (ID# 2022-22-FR). Briefly, I278T mice (n = 3 M+3F each cohort) at 6 weeks of age were switched from a standard extruded diet to amino-acid defined diets containing different amount of Met (Envigo RMS, France). Specifically, the custom-formulated isocaloric and isonitrogenous diets contain 0.5, 2.0, 4.0 and 6.0 g/kg Met (TD.220655, TD.220656, TD.220657 and TD.220658, respectively) following a similar formulation as used previously [15] with the diet containing 4.0 g/kg Met approximately matching sulfur amino acids content of a standard mouse chow. A single cohort of WT mice (n = 3 M+3F) was set up on 4.0 g/kg Met diet and served as a control group. After two weeks on the diets, mice were euthanized by CO_2_ asphyxiation, perfused using 1x phosphate-buffered saline (PBS) with their biological samples (blood, liver, kidney and brain) harvested and stored at −80 °C.

Another study using solely I278T mice was designed to assess the impact of biological reductants on plasma thiol homeostasis and efficacy of PEG-CBS. This study was conducted at the University of Colorado Anschutz Medical Campus under the IACUC-approved protocol# B-49414(03)1E. Briefly, I278T mice were randomized into four cohorts each consisting of two males and two females (n = 4 each) representing vehicle (PBS)-injected controls and groups of mice each receiving a different biological thiol-based reductant: 1000 mg/kg/day N-acetylcysteine (NAC), 1000 mg/kg/day 2-mercaptoethane sulfonate (MESNA) or 200 mg/kg/day cysteamine (CA). The test substances or vehicle were administered daily for five days intraperitoneally split into two doses each day (executed at 7AM and 7PM). After ten days recovery period back to the initial metabolite levels, similar regimen was executed again now accompanied with an administration of PEG-CBS to assess potentially synergistic effect of thiol-based reductants and ERT for HCU. Treatment with PEG-CBS preceded administration of thiol-based reductants by one day just to have an initial efficacious CBS activity in plasma before the first dose of reductants. PEG-CBS was administered at the dose of 8 mg/kg/day in a single subcutaneous injection each day at 9AM for a period of five days (i.e. until PEG-CBS reaches steady-state plasma levels in mice [16]). Blood samples were collected on day 1 (D1, baseline), D5 & D8 (initial & steady-state efficacy of thiol-based reductants alone), D15 (recovery) and D19 & D22 (initial & steady-state efficacy of thiol-based reductants co-administered with PEG-CBS). Heparinized plasma was prepared after collecting blood from submandibular vein into Capiject T-MLHG lithium heparin (12.5 IU) tubes with gel (Terumo Medical Corporation). The tubes were then centrifuged at 1,200×*g* for 10 min, followed by transfer of plasma into 1.5 ml tubes and storage at −80 °C.

**Metabolomics.** Panel of sulfur amino acid metabolites including tHcy, total Cys (tCys), Met and Cth were determined by stable-isotope-dilution liquid chromatography tandem mass spectrometry (LC–MS/MS) as described elsewhere [20]. In addition, protein-unbound fractions of Hcy were determined after precipitation of plasma proteins by 20-fold excess of acetonitrile and clarification of the resulting supernatant by centrifugation at 17,000×*g* at room temperature for 10 min.

**Biothiol quantification by the multimodal fluorescent Probe 1**. Quantitative determination of tHcy and tCys using Probe 1 was performed as described previously with a few modifications [17]. Plasma tHcy and tCys as well as standards (0–1000 μM for Hcy and 0–500 μM for Cys) were diluted with equal volumes of 10 mM Tris(2-carboxyethyl)phosphine (TCEP) and incubated for 20 min while constantly shaking at 37 °C. Reduced samples and standards (2–5 μl) were transferred into a black 96-well plate and mixed to a final volume of 200 μl with detection buffer containing 1x PBS, 10 μM Probe 1 and either 70 % or 40 % DMSO for detection of tHcy or tCys, respectively. The volume of standards and the samples taken into the assay were adjusted based on the expected concentrations. For example, plasma from HCU mice was typically assayed after 100-fold dilution, while cell culture samples were usually diluted 40-fold. Probe 1 was allowed to bind biothiols for 30 min at 37 °C. For Hcy quantification the detection buffer was further supplemented with 10 μM TCEP. Fluorescence was assessed at Ex/Em 488 nm/542 nm for Hcy and 360 nm/453 nm for Cys detection. The protein unbound fraction of biothiols was determined after precipitation with equal volumes of 0.4 M perchloric acid (PCA) followed by centrifugation at 17,200 rpm, 4 °C, 20 min. For kinetic assessment of the action of PEG-CBS, samples were collected at designated timepoints and mixed in equal volumes with the denaturing solution containing 10 mM TCEP and 1 % SDS and processed as described above. Note that Probe 1 can also be used for quantification of GSH as described in the original report [17]. However, the detection of GSH is slightly affected by Cys due to partially overlapping fluorescent signals. Therefore, we decided not to use Probe 1 for quantification of GSH in our study.

**CBS activity assay**. CBS activity was quantified using methylene blue assay as described previously [21]. Briefly, the assay was performed in a reaction buffer containing 100 mM Tris-HCl pH 8.6 and 0.2 mM PLP. Hcy, Cys and the studied thiol-based reductants NAC, MESNA and CA serving as putative CBS substrates were mixed in the reaction buffer and catalytic reaction was initiated by addition of CBS45 to a final concentration of 12.5 μg/ml. The reaction proceeded for 15 min at 37 °C with constant shaking after which 50 μl of the mixture was added to a 96-well plate with equal volumes of 4 % zinc acetate solubilized in 1.2 N HCl to trap the generated H_2_S. The plate was sealed, incubated on ice for 15 min and subsequently reacted with 15 μl of a solution containing 30 mM N,N-dimethyl-*p*-phenylenediamine sulfate and 45 mM ferric chloride. Methylene blue, the product of this reaction, was measured spectrophotometrically at 670 nm and CBS-catalyzed H_2_S production was quantified using a serial dilution of Na_2_S as a standard.

Activity assessment of various CBS constructs before and after PEGylation was performed using radiometric assay as described previously [18,22]. Briefly, diluted protein or PEGylation reaction mixture (420 ng) was assayed in a 100 μl reaction volume for 30 min at 37 °C in the presence of 10 mM serine. The reaction was initiated by addition of 10 mM Hcy and was terminated by an immediate cooling of the mixture in ice water and the labeled product was separated from the substrates by paper chromatography overnight. Spots corresponding to Cth were cut-out and radioactivity was determined by using a scintillation counter.

**Cell culture**. HepG2 cells (ATCC) were grown in DMEM supplemented with 10 % heat-inactivated fetal bovine serum and 1 % penicillin/streptomycin mixture. Cells were seeded into 6-well plates and allowed to adhere overnight. CBS45 was supplied at a final concentration of 25 ng/μl either directly to the media or during medium exchange. For the detection of cellular Hcy production, 20 μl of media were taken at the designated time points and mixed with equal volumes of a 1 % SDS and 10 mM TCEP for quantification by Probe 1 as described above.

***In vitro* release of biothiols by reductants**. Plasma from several I278T mice was pooled, mixed with stock solutions of NAC, MESNA, CA, ascorbic acid (Vitamin C) or 6-hydroxy-2,5,7,8-tetramethylchroman-2-carboxylic acid (Trolox) to yield final concentrations of 0–3 mM and incubated at 37 °C for 20 min. Subsequently, reduced plasma was precipitated by equal volumes of 0.4 M PCA, and unbound fraction was quantified as described above.

**Statistics**. All data are presented as the mean ± SD unless otherwise stated. When comparing multiple different experimental arms, data were analyzed by ANOVA, followed by Tukey's multiple comparisons test, unless stated otherwise. A p-value of <0.05 was considered significant (marked by asterisk in the figures).

## 3 Results

### 3.1 Probe 1 provides a quick and reliable quantification of Hcy and Cys in biological samples

**Fig. 1:**
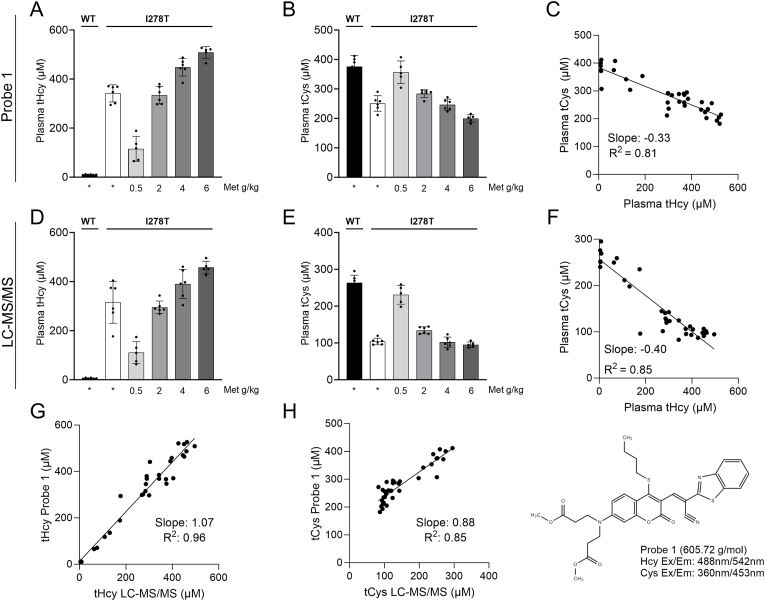
**Validation of Probe 1 for quantification of homocysteine and cysteine in biological samples.** The tHcy (**A, D**) and tCys (**B, E**) were quantified in plasma of I278T mice fed an amino acid-defined diet with different amount of Met using either the fluorescent Probe 1 (10 μM final concentration in the assay; **A, B**) or LC-MS/MS (**D, E**) as a current standard. Samples were measured as single values (LC-MS/MS) or technical triplicates (Probe 1) and presented as means ± SD (n = 5–6). Panels **C** and **F** show correlation between plasma tHcy and tCys as determined by Probe 1 and LC-MS/MS, respectively. Panels **G** and **H** show correlation between Probe 1 and LC-MS/MS quantification of tHcy and tCys, respectively. Inset in the bottom right corner shows structure and basic properties of Probe 1.

Fig. 1 shows a comparative evaluation a novel multimodal fluorescent probe (Probe 1) for quantification of tHcy and tCys against a standard LC-MS/MS method. For this purpose, we used plasma samples from I278T mice fed with amino acid-defined diets providing different amounts of Met (0.5, 2, 4 or 6 g/kg Met) and from healthy WT mice fed with 4 g/kg Met diet resembling the sulfur amino acid content of a standard mouse chow, which served as controls. Based on our previous experience using a different HCU mouse model [15], we anticipated gradual differences in plasma tHcy and tCys. Fig. 1A shows that compared to WT controls, all I278T cohorts had significantly and substantially elevated plasma tHcy, which showed anticipated gradual differences based on Met intake. Specifically, using Probe 1, mean plasma tHcy amounted to 115, 334, 447 and 507 μM in I278T mice on 0.5, 2, 4 and 6 g/kg Met diet, respectively. Fig. 1B shows similar gradual differences in plasma tCys in I278T mice on diets with adjusted Met content. Specifically, using Probe 1, mean plasma tCys was 356, 284, 246, 199 μM in I278T mice on 0.5, 2, 4 and 6 g/kg Met diet, respectively. Interestingly, the diet with the lowest Met content of 0.5 g/kg resulted in a normalization of plasma tCys levels similar to WT controls (376 μM). Fig. 1C illustrates a negative correlation of plasma tHcy and tCys in I278T mice on Met-adjusted diets as determined by Probe 1 yielding a coefficient of determination of 0.81 and a regression coefficient of −0.33.

Fig. 1D and E shows the quantification of plasma tHcy and tCys in the same plasma samples as used in Fig. 1A and B, but using a standard LC-MS/MS method. Specifically, mean plasma tHcy amounted to 111, 295, 390, 457 μM in I278T mice on 0.5, 2, 4 and 6 g/kg Met diet, respectively (Fig. 1D), and mean plasma tCys was 230, 134, 102, 95 μM in I278T mice on 0.5, 2, 4 and 6 g/kg Met diet, respectively (Fig. 1E). Fig. 1F illustrates a negative correlation of plasma tHcy and tCys in I278T mice on Met-adjusted diets as determined by using standard LC-MS/MS approach yielding a coefficient of determination of 0.85 and a regression of −0.40.

Fig. 1G illustrates a fundamental agreement of the measured values (R^2^ = 0.956) determined by the Probe 1 method or LC-MS/MS approach, although the fluorescent quantification of tHcy overestimated the absolute values by 7 %. The coefficient of determination for tCys is 0.85 (Fig. 1H). Thus, the fluorescence approach utilizing multimodal Probe 1 represents a simple, reliable, time-saving and cost-effective method to quantify biothiols in biological samples yielding comparable results to the state-of-the-art LC-MS/MS approach requiring expensive instrumentation and a skilled operator.

### 3.2 CBS efficacy is limited by the availability of substrates Ser and reduced Hcy

**Fig. 2:**
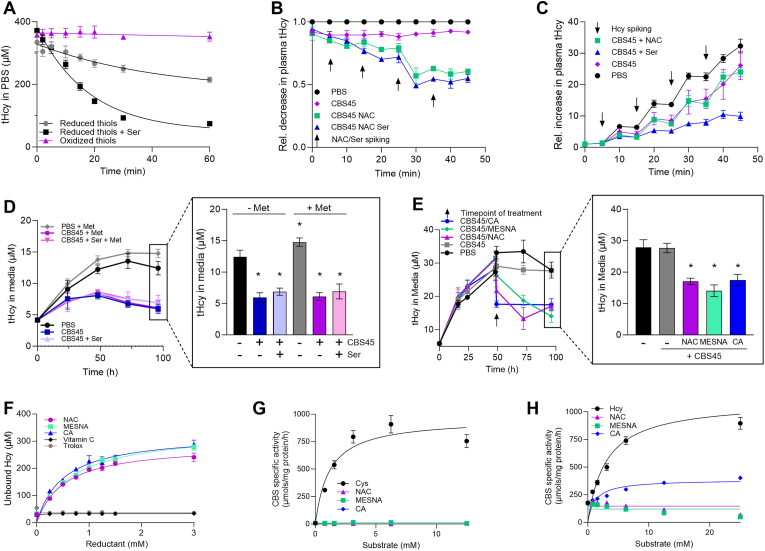
**Impact of reductant on Hcy availability and efficacy of CBS to degrade it *in vitro*. (A)** To mimic concentrations found in HCU plasma, PBS was supplemented with 120 μM Ser and either reduced thiols (360 μM Hcy, 130 μM Cys; grey) or their equimolar oxidized disulfide forms (180 μM homocystine, 65 μM cystine; purple). Additional 500 μM Ser was added to one set of samples containing the reduced thiols (black). Subsequently, 25 ng/μl CBS45 was added and Hcy elimination was monitored using Probe 1 (10 μM). **(B)** Pooled plasma from I278T mice (black) was supplemented with 25 ng/μl CBS45 alone (purple) or in combination with 400 μM NAC (lime) or mixture containing 400 μM NAC and 400 μM Ser (blue). NAC or NAC + Ser combination was repeatedly added as indicated by the arrows. Data are displayed as relative means ± SD (n = 4). **(C)** Similarly, pooled plasma from WT mice (black) was supplemented with 25 ng/μl CBS45 alone (purple) or in combination with 400 μM NAC (lime) or 400 μM Ser (blue). Addition of 400 μM Hcy in 10 min increments is indicated by the arrows. Concentration of tHcy were determined by using Probe 1 (10 μM). Data are displayed as relative means ± SD (n = 4). **(D)** HepG2 cells were seeded at the density of 300,000 cells per well into a 6-well plate. Standard media or media containing additional 2 mM Met were supplemented with PBS for controls (black, grey), 25 ng/μl CBS45 (blue, purple) or 25 ng/μl CBS45 in combination with 1 mM Ser (light blue, pink). The tHcy in media was quantified by using Probe 1 (10 μM) at designated timepoints with the inset zooming in at the last time point after 96 h. **(E)** HepG2 cells were seeded at the density of 600,000 cells per well into a 6-well plate and were incubated untreated for 48 h to generate a substantial fraction of oxidized Hcy (homo-/hetero-disulfides, protein-bound). Thereafter (indicated by the arrow), media were supplemented with equal volumes of PBS (black), 25 ng/μl CBS45 (grey) or combination of 25 ng/μl CBS with 2 mM NAC (purple), MESNA (lime) or CA (blue). The tHcy in media was quantified by using Probe 1 (10 μM) at designated timepoints with the inset zooming in at the last time point after 96 h (48 h after the treatment). Data are displayed as mean ± SD (n = 4) and were statistically analyzed by ordinary one-way ANOVA with Tukey's multiple comparison test against PBS controls. **(F)** Pooled I278T plasma was treated with different concentrations (0–3 mM) of NAC (purple), MESNA (lime), CA (blue), Vitamin C (black) and Trolox (grey). Subsequently, samples (n = 4 each group) were precipitated and protein-unbound Hcy was quantified in the clarified supernatants by using Probe 1 (10 μM). **(G)** Reductants NAC (purple), MESNA (lime) and CA (blue) were tested for their ability to compete with Cys as the first substrate forming PLP-aminoacrylate adduct in CBS catalytic site. The CBS specific activity was determined by methylene blue assay quantifying generated H_2_S as a common by-product of CBS-catalyzed condensations. Data represent means ± SDs (n = 4 each group). **(H)** Similarly, reductants NAC (purple), MESNA (lime) and CA (blue) were tested for their ability to compete with Hcy as the second substrate attacking PLP-aminoacrylate adduct using Cys as the first substrate. The CBS specific activity was determined by methylene blue assay quantifying generated H_2_S as a common by-product of CBS-catalyzed condensations (n = 4 each group). Data are presented as mean ± SD (n = 4 each group).

Having established a simple, fast and reliable assay to quantify biothiols, we decided to gain insight into the mechanism of action of CBS-based ERT using *in vitro* and cell-based approach to determine whether the modulation of biothiol homeostasis by the biological reductants affects its efficacy. Fig. 2A shows that only reduced Hcy can serve as a substrate for CBS. Specifically, we simulated metabolic environment found in HCU plasma by supplementing 1x PBS pH 7.4 with 120 μM Ser and either reduced or oxidized forms of Hcy and Cys, i.e. 360 μM Hcy with 130 μM Cys or 180 μM homocystine (Hcy-Hcy) with 65 μM cystine (Cys-Cys). While there was no decrease of Hcy observed in the presence of oxidized thiols, reduced thiols resulted in a gradual depletion of Hcy in the presence of 25 ng/μl CBS45 (i.e. steady-state plasma concentration of pegtibatinase found efficacious in mouse models of HCU [16]). The CBS-catalyzed degradation of Hcy increased by 2.3-fold when concentration of CBS co-substrate Ser was increased 4.2-fold to 500 μM suggesting that Ser availability limits the efficacy of CBS to degrade Hcy.

To validate our initial observation, we added CBS45 into the plasma from HCU mice and observed only a minor (∼10 %) drop in tHcy concentration, which did not change over time (Fig. 2B). This observation suggests that the reduced (available) Hcy was consumed immediately and the oxidized Hcy (disulfide or protein-bound) remained inaccessible. However, a gradual decrease in tHcy levels was observed after the HCU mouse plasma supplemented with CBS45 was repeatedly spiked with the reductant NAC alone or in combination with CBS co-substrate Ser (Fig. 2B). Such treatments eventually resulted in approximately 40 % and 50 % decrease of tHcy concentration within 30 min follow-up in case of NAC alone and in combination of NAC and Ser, respectively. These results confirm that the availability of reduced Hcy in HCU plasma strongly limits the efficacy of CBS, and supplementation with Ser can lead to a further potentiation of CBS efficacy.

Similar conclusion can be drawn from the experiment shown in Fig. 2C, where WT mouse plasma was repeatedly spiked with reduced Hcy in the absence or presence of CBS45. CBS45 produced an approximately 20 % decrease of tHcy concentrations after each Hcy spike. While NAC supplementation did not further stimulate Hcy degradation by CBS45, Ser supplementation resulted in an additional approximately 70 % decrease of tHcy concentrations (Fig. 2C). These results again point to Ser availability as a limiting factor for CBS efficacy in degrading Hcy. Furthermore, it is also conceivable that a certain portion of reduced Hcy may get oxidized (by either forming homo or mixed disulfides or binding to Cys protein residues) and thus becoming unavailable as a substrate for CBS.

### 3.3 Exported reduced Hcy serves as a substrate for CBS

Next, we utilized a cell-based system to assess the ability of CBS to degrade exported Hcy. We used HepG2 cells derived from liver, since the liver serves as the main Met-metabolizing and Hcy-producing organ. Fig. 2D shows that proliferating HepG2 cells produce and export Hcy into culture medium at linear rate for up to 48 h after which the tHcy concentration in the media did not change. Supplementation with 2 mM Met (i.e. 10-fold excess compared to 0.2 mM Met in normal culture medium) resulted in a small, but significant 19 % increase in tHcy concentration in the medium. Addition of 25 ng/μl CBS45 into the culture medium resulted in a 52 % and 51 % decrease of tHcy concentrations compared to the respective controls in normal and Met-spiked medium, respectively (Fig. 2D inset). Supplementation with 1 mM Ser (i.e. 2.5-fold excess compared to 0.4 mM Ser in normal culture medium) did not have any significant impact on CBS-mediated Hcy degradation in the culture medium. These results suggest that cells export Hcy in its reduced form and CBS, present in the culture medium, can efficiently degrade it.

Fig. 2E illustrates that seeding twice more HepG2 cells than in experiment shown in Fig. 2D results in doubling of tHcy concentration in the medium over period of 48 h. We expected that the exported reduced Hcy will be oxidized either forming disulfides or binding to Cys residues of serum proteins in the medium, which would not act as a substrate for CBS. Indeed, delayed addition of CBS45 to the medium (at the 48 h time point in this experiment) did not decrease tHcy levels in the media after an additional 48 h treatment period. However, when the delayed treatment with CBS45 was combined with the reductants, specifically NAC, MESNA and CA (2 mM each), the Hcy-degrading activity of CBS45 was restored, resulting in a significant 39 %, 50 % and 37 % decreases of tHcy concentration in the media, respectively (Fig. 2E inset). These results indicate that the reduced Hcy, after it exits the cells and enters the culture medium, gets quickly oxidized (thus becoming unavailable as a substrate for CBS), but its oxidation can be partially prevented this process can be partially rescued by the addition of biological reductants, such as NAC, MESNA or CA.

### 3.4 Thiol-based reductants liberate Hcy and Cys from proteins

As discussed above, a certain pool of Hcy may bind to proteins and as such may become unavailable for CBS-mediated degradation. To assess the efficacy of biologically compatible reducing agents liberating protein-bound Hcy, we screened five biological reductants for their capacity to reduce and release Hcy from plasma proteins: the thiol-based reductants NAC, MESNA and CA and Trolox and ascorbic acid, which are known to donate electrons through their respective hydroxyl groups. Fig. 2F shows that NAC, MESNA and CA, but not Trolox and ascorbic acid, liberated Hcy from the protein of mouse HCU plasma. The efficacy of each of these three compounds was comparable and each exerted its effect in a concentration-dependent manner. Specifically, the highest tested (3 mM) concentration of NAC, MESNA and CA liberated 40 %, 46 % and 48 % of Hcy, respectively, into a protein-unbound fraction from the mouse HCU plasma containing 608 μM tHcy. Trolox and vitamin C did not show any impact on release of protein-bound Hcy. Therefore, further evaluation was carried out only using thiol-based reductants NAC, MESNA and CA.

As Cys serves as an alternative substrate of CBS replacing Ser and producing hydrogen sulfide (H_2_S) [23], we next investigated whether NAC, MESNA and CA can compete with Cys or Hcy as alternative CBS substrates. We monitored the production of H_2_S, which represent a common side product in addition to various thioethers for all the mentioned substrates and reactions. Fig. 2G shows that neither NAC, MESNA nor CA can replace Cys in its function to form adducts similar to aminoacrylate with a PLP cofactor of CBS. However, as Fig. 2H illustrates, CA, but not NAC or MESNA, was able to partially replace Hcy in its function to attack PLP-aminoacrylate adduct and thus enabled a catalytic cycle. This result indicates that, while NAC and MESNA would not compete with the substrates of CBS, CA could partially impair the ability of CBS to degrade Hcy.

### 3.5 Thiol-based reductants increase the efficacy of PEG-CBS in I278T mice

**Fig. 3:**
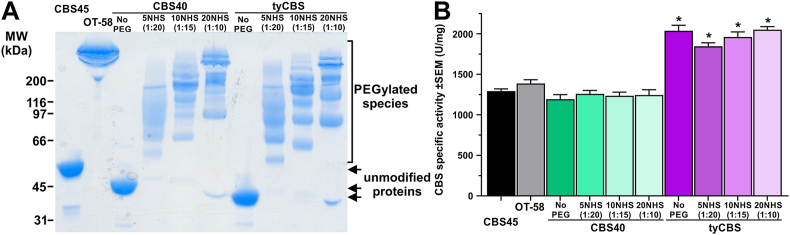
**Preparation of PEG-CBS.** Three linear 5, 10, and 20 kDa NHS ester PEGs (5NHS, 10NHS, and 20NHS) were used to modify human CBS40 and yeast tyCBS in a variable molar excess of PEG to CBS subunit as indicated in the figure. Newly generated functional analogs of pegtibatinase were compared to a previously evaluated unmodified CBS45 and its PEGylated version OT-58 (also known as pegtibatinase). (**A**) Unmodified enzymes and their PEGylated conjugates were resolved on a 10 % Miniprotein TGX SDS-PAGE (10 μg/lane) and stained with Safe Stain (Invitrogen) according to manufacturer's recommendation. The arrow points to subunits of unmodiﬁed enzymes, while the bracket encompasses the region where their PEGylated conjugates migrated. (**B**) Unmodified enzymes and their PEGylated conjugates were assayed using radiometric CBS activity assay and compared. Bars represent average from four separate measurements and error bars designate SEMs. Asterisks (∗ = p < 0.05) designate a significantly increased CBS specific activity of tyCBS compared to CBS45.

Next, we evaluated potential benefits of thiol-based reductants in reducing plasma tHcy levels in mouse model of HCU either alone or in combination with CBS-based ERT. For this purpose, we generated a functional analog of pegtibatinase, which we have extensively characterized in our previous work [[12], [13], [14], [15], [16]]. We explored two options: a further optimized human CBS40, and heme-independent tyCBS, which were PEGylated with either 5, 10 or 20 kDa linear N-hydroxysuccinimide ester (NHS)-activated methoxyPEG (Fig. 3). PEGylation was performed to increase the half-life of the ERT in circulation and potentially to reduce immunogenicity as shown previously for pegtibatinase [10]. Although PEGylation has not been fully optimized, as apparent from multiple bands corresponding to CBS subunits modified with variable number of PEG moieties compared to pegtibatinase (Fig. 3A), variable PEGylation does not substantially affect biological properties, most notably half-life, as long as at least one PEG moiety is attached to CBS [11,16]. The tyCBS is the smallest out of the studied enzymes as it does not possess a heme-binding domain and does not require heme for its catalytic function. In addition, tyCBS has about 60 % higher CBS specific activity than human CBS45 and CBS40 and, similarly to the human options, its activity is not significantly impaired by PEGylation (Fig. 3B). To evaluate a non-human functional analog of pegtibatinase with a higher CBS specific activity, we decided to continue with tyCBS modified with 20NHS PEG, further designated as PEG-CBS.

**Fig. 4:**
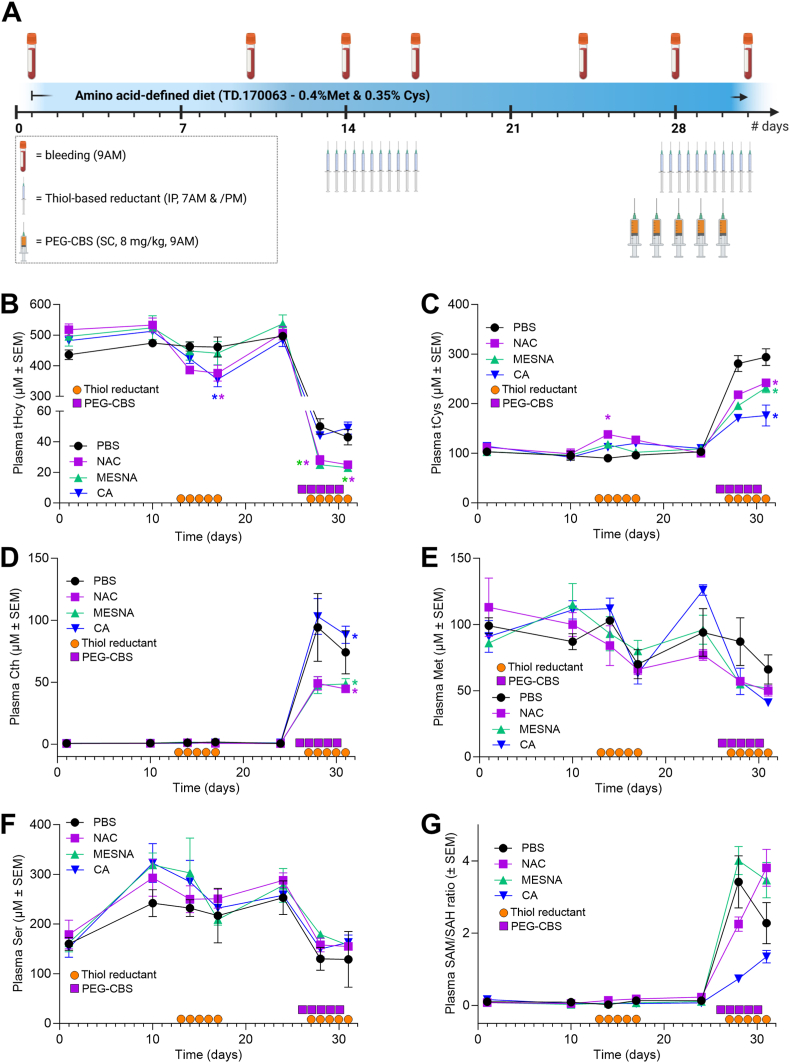
**Evaluation of the thiol-based reductants in I278T mice.** Four I278T mouse cohorts (n = 6–8 each) were acclimated to the amino acid-defined diet containing 0.4 % Met and received vehicle (PBS, black), NAC (purple), MESNA (lime) or CA (blue) alone and after a washout period received the same in combination with PEG-CBS as described in detail in the main text. The orange circles and purple squares denote administration days of PBS or reductants via intraperitoneal injections and PEG-CBS via subcutaneous injections, respectively. **(A)** Schematics of a mouse study. Plasma tHcy **(B)**, tCys **(C)**, Cth **(D)**, methionine **(E)**, serine **(F)** and SAM/SAH ratio **(G)**. Color-coded asterisks denote significance (∗ = p < 0.05).

Fig. 4A illustrates the design of a single comprehensive mouse study evaluating NAC, MESNA and CA alone or in combination with PEG-CBS in the same animal as enabled by a washout period in between treatment periods (see Material and Methods for more details). Fig. 4B shows the effect of the three studied thiol-based reductants alone and in combination with PEG-CBS on plasma tHcy levels. After 5 days of treatment, we observed a significant 28 % and 26 % decrease after treatment with NAC and CA, respectively (D10 vs D17), while treatment with MESNA only showed non-significant 11 % drop in plasma tHcy levels. Co-administration of PEG-CBS resulted in a substantial and significant decrease of plasma tHcy levels in all cohorts. While PEG-CBS administration resulted in tHcy decrease by 91 % and 90 % in PBS- and CA-injected I278T mice, respectively (D24 vs D31), mice receiving NAC and MESNA showed even bigger and significantly different drop (i.e. by 95 % and 96 %, respectively). Specifically, tHcy concentration dropped to 43 and 49 μM in PBS and CA cohorts, respectively, while NAC and MESNA cohorts produced on average 25 and 23 μM tHcy, respectively. Thus, administration of NAC and CA in their own can significantly decrease plasma tHcy levels. Moreover, NAC and MESNA can increase the efficacy of PEG-CBS therapy, so that the combination of either of these reductants with PEG CBS can reduce plasma tHcy in I278T mice to near-normal levels (∼15 μM in WT mice on the same diet [14]).

Fig. 4C shows the effect of thiol-based reductants alone and in combination with PEG-CBS on plasma tCys levels. After 5 days of treatment, we observed a significant 14 % increase only after the treatment with NAC (D10 vs D17). Co-administration of PEG-CBS resulted in an anticipated substantial and significant increase of plasma tCys levels in all cohorts. While PEG-CBS administration resulted in a complete normalization in PBS-injected cohort (294 μM, D24 vs D31), its elevation was less pronounced in cohorts receiving thiol-based reductants. Specifically, plasma tCys increased to 242 and 232 μM in NAC- and MESNA-treated mice, while CA only increased tCys to 176 μM. Taken together, NAC alone was able to slightly increase low plasma tCys levels observed in I278T mice. However, all studied thiol-based reductants significantly reduced efficacy of PEG-CBS to improve plasma tCys levels. While co-administration of NAC or MESNA with PEG-CBS still resulted in a doubling of the initial levels, CA apparently interfered with plasma tCys normalization.

Fig. 4D shows effect of thiol-based reductants alone and in combination with PEG-CBS on plasma Cth levels. As anticipated, thiol-based reductants alone did not affect plasma Cth levels. However, marked and significant elevation of plasma Cth levels was observed after co-administration of PEG-CBS. Interestingly, I278T mice receiving PBS or CA showed significantly higher plasma Cth levels compared to those on NAC and MESNA. Specifically, plasma Cth concentrations increased to 74 and 88 μM in PBS and CA cohorts, respectively (D24 vs D31), while the mice on NAC and MESNA achieved 45 and 49 μM Cth, respectively. Taken together, plasma Cth levels increased only after administration of PEG-CBS as a marker of PEG-CBS activity and efficacy. Interestingly, the difference in plasma Cth levels between NAC- and MESNA-treated cohorts compared to PBS- and CA-treated ones correlates with the similar pattern observed for plasma tHcy levels (Fig. 4B).

Fig. 4E shows effect of thiol-based reductants alone and in combination with PEG-CBS on plasma Met levels. Thiol-based reductants alone did not show any significant effect on plasma Met levels despite apparent downward trend. Co-administration of PEG-CBS resulted in a significant decrease of plasma Met concentrations in all cohorts by 30 %, 36 %, 46 % and 68 % to 66, 50, 52 and 41 μM Met concentrations in cohorts injected with PBS, NAC, MESNA and CA, respectively, which essentially resulted a normalization of plasma Met levels (∼50 μM in WT mice on the same diet [14]). Taken together, PEG-CBS alone or in combination with the thiol-based reductants significantly decreased and essentially normalized plasma Met concentrations in I278T mice.

Fig. 4F shows effect of thiol-based reductants alone and in combination with PEG-CBS on plasma Ser levels. Interestingly, the amino acid-defined diet mimicking the standard mouse chow resulted in on average 80 % increase in plasma Ser concentrations compared to the standard chow (D10 vs D1). Thiol-based reductants alone did not show any significant impact on plasma Ser levels, but administration of PEG-CBS resulted in on average 44 % decrease in all cohorts. There was no significant difference between PEG-CBS administered alone or in combination with the thiol-based reductants.

Fig. 4G shows the effect of thiol-based reductants alone and in combination with PEG-CBS on plasma SAM/SAH ratio (also known as the methylation index). Accumulation of SAH in HCU substantially impacts methylation index of untreated I278T mice showing more than one order of magnitude lower SAM/SAH ratios compared to WT mice (e.g. 0.16 versus 4.76 [13]). Thiol-based reductants alone did not improve this imbalance. However, all groups experienced a dramatic increase in their SAM/SAH ratios after the administration of PEG-CBS. However, no significant differences were found in the SAM/SAH ratios between the PEG-CBS treatment alone (PBS cohort) and in combination with the thiol-based reductants (NAC, MESNA and CA cohorts).

### 3.6 Thiol-based reductants replace Hcy from plasma proteins *in vivo*

**Fig. 5:**
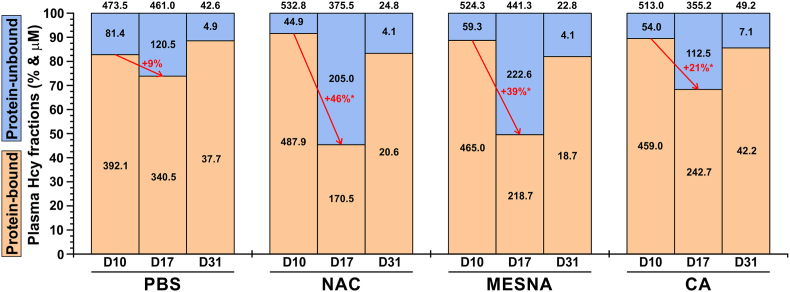
**Distribution of plasma Hcy fractions during evaluation of thiol-based reductants in I278T mice.** Plots display relative proportions of protein-bound (light brown) and protein-unbound (light blue) Hcy of plasma tHcy pool (representing 100 %) at the baseline (D10), after the treatment with thiol-based reductants (D17) and after the co-administration of PEG-CBS and thiol-based reductants (D31) in I278T cohorts designated as PBS (control), NAC, MESNA and CA. Black numbers within and above the bars correspond to average concentrations in protein-bound/unbound Hcy and plasma tHcy, respectively. Red color and arrows designate a change in relative proportion of protein-bound versus protein-unbound plasma Hcy with a significance of the change (∗ = p < 0.05).

To further understand the mechanism which underlies the ability of NAC and MESNA to increase the efficacy of PEG-CBS to degrade plasma Hcy (Fig. 4B), we analyzed proportion of protein-unbound Hcy compared to tHcy in plasma samples when the mice got acclimated to the amino acid-defined diet (D10), after 5 days of treatment with thiol-based reductants (D17) and at steady-state levels of PEG-CBS when co-administered with the thiol-based reductants (D31). Both plasma tHcy and protein-unbound Hcy were determined experimentally, while protein-bound Hcy was calculated as a difference between tHcy and protein-unbound Hcy. Fig. 5 shows that all three evaluated reductants significantly increased the proportion of protein-unbound Hcy in plasma compared to the pre-treatment period. Specifically, administration of NAC and MESNA resulted in a 46 % and 39 % increase of protein-unbound Hcy at D17, respectively, while administration of CA increased the proportion of protein-unbound Hcy only by 21 % compared pre-treatment baseline (D10). Interestingly, when co-administered with PEG-CBS, which by itself resulted in >90 % decrease of plasma tHcy (Fig. 4B), the distribution of Hcy has been restored to the pre-treatment levels. Taken together, the analysis of Hcy fractions in plasma lend a support to our hypothesis that the thiol-based reductants, particularly NAC and MESNA, markedly increase proportion of protein-unbound Hcy. Despite normalization of Hcy distribution after the co-administration of PEG-CBS and thiol-based reductants, the presence of NAC or MESNA potentiated or acted synergistically with PEG-CBS as evidenced by the plasma tHcy dropping to ∼24 μM levels compared to ∼46 μM concentration in the PBS- and CA-injected I278T cohorts (Fig. 4B).

## 4 Discussion

Strict adherence to a Met-restricted diet supplemented with Met-free amino acid formula and often combined with betaine administration currently represents the only available treatment option for severe pyridoxine non-responsive form of HCU [8]. Recent advances in novel therapeutic approaches based on enzymatic degradation of Hcy or its precursor Met hold great promise for the HCU community [24]. The most advanced project and currently the only one in active phase of development is pegtibatinase, ERT for HCU based on truncated human CBS, which showed impressive efficacy in preclinical studies decreasing plasma tHcy by up to 90 % in multiple mouse models of HCU [12,14,16] and which recently entered Phase 3 clinical evaluation. Using novel tools, such as the multimodal fluorescent Probe 1, which permits quick and reliable quantification of biothiols Hcy and Cys, here we aimed to gain further insights into the mechanism of action of CBS-based ERT and explored a novel concept of a reductant-based approach aimed to increase its efficacy.

Accumulation of Cth in plasma after the treatment of HCU mice with pegtibatinase in previous studies [12,14,16] or PEG-CBS in this study (Fig. 4D) clearly indicates CBS-catalyzed degradation of Hcy. However, it was unclear how CBS accesses its co-substrate Hcy, which in plasma is rarely found in its reduced form (∼1 %). Hcy preferentially conjugates Cys sulfhydryl residues of plasma proteins (∼70–80 %) and also forms homo- and hetero-disulfides with other thiols, mostly Cys (∼20–30 %) [8,25,26]. However, this balance is dramatically disturbed in HCU, where free reduced Hcy linearly correlates with the tHcy, it remained less than 10 % for up to 180 μM tHcy, but increased up to ∼35 % for tHcy above this threshold [8], still with the overwhelming majority in the oxidized forms (i.e. protein-bound or disulfides). Due to its high pKa, less than 1 % of Hcy sulfhydryl groups undergo proton dissociation when it is released from the tissues into the systemic circulation. However, given the high nucleophilicity of the Hcy thiolate anion, it can rapidly displace Cys from albumin through a series of disulfide isomerization, leading to its depletion in plasma [25,27]. In this context, we tested whether CBS in plasma is capable of degrading progressively increasing amounts of Hcy before it binds to plasma proteins. Although the CBS co-substrate Ser was found a rate-limiting factor in our *in vitro* study, we have found that the turnover rate of CBS45 sufficiently supported removal of the spiked Hcy before it could conjugate with plasma proteins (Fig. 2C). Further support for this conclusion came from our cell-based assay using HepG2 cells (Fig. 2D). During their exponential growth, hepatocytes produce and export Hcy, possible due to an increased transmethylation rate during cell division [28], which plateaus out when the cells reach confluency (Fig. 2D and E). Here we demonstrated that CBS45 in the medium of growing HepG2 cells is able to degrade most of the generated and exported Hcy under both the standard and Met-loading conditions (up to 50 %). Despite being found a rate-limiting factor in our *in vitro* plasma study, Ser content was not limiting in our cell-based study. Indeed, Ser levels can be easily replenished by several metabolic interconversions [29,30] and, therefore, are unlikely to represent a limiting factor for CBS ERT *in vivo*.

CBS was unable to use the oxidized forms of Hcy as a substrate (Fig. 2A). Indeed, when incubated with HCU plasma from I278T mice, CBS was able to rapidly metabolize only the free, reduced Hcy fraction (Fig. 2B). Further support came from our cell culture experiment, where we have observed that when Hcy was accumulated and oxidized in the medium, addition of CBS did not change tHcy concentrations (Fig. 2E). The results presented here demonstrate that a novel, efficient way to promote CBS-catalyzed Hcy degradation can be achieved by modulating redox status of Hcy, i.e. supplementation with biological reductants. We have evaluated three thiol-based reductants (NAC, MESNA and CA). These are FDA-approved drugs to treat paracetamol overdose [31], to be used as a chemotherapy adjuvant [32] and to treat cystinosis [33], respectively. NAC was previously shown to lower both mildly elevated [34,35] and severely elevated plasma tHcy levels in I278T mice [36]. Specifically, when provided as 40 mM solution in drinking water, NAC raised plasma tCys by ∼50 % (which was still just around 54 % of WT controls) and decreased plasma tHcy by ∼13 % compared to I278T on normal water. In our study, intraperitoneal administration of 1000 mg/kg/day NAC alone resulted only in approximately 14 % increase of plasma tCys, but yielded a ∼27 % decrease of plasma tHcy (Fig. 4B and C). NAC is typically thought as a bioavailable prodrug of Cys to boost biogenesis of GSH [37]. Thus, part of the antioxidant effect of NAC may be indirect and mediated by GSH. However, NAC is also capable of breaking thiolated proteins, thus releasing free thiol and reduced protein. Interestingly, both the released thiol (typically Cys under normal conditions) and the reduced protein (typically mercaptoalbumin) have better direct antioxidant activity than NAC [37]. In HCU, the released thiol is predominantly Hcy, which can be then metabolized by CBS-based ERT as shown in the present study. Notably, dietary supplementation of Cys did not have any impact on plasma tCys and tHcy levels in I278T mice [15]. Furthermore, according to the guidelines for management of HCU [8], there is no evidence that dietary supplementation of l-cystine improves clinical outcome of HCU patients. Therefore, further research and clinical studies are needed to clarify the clinical benefit of l-cystine supplementation.

The direct use of GSH as a biological reductant, alongside NAC, MESNA, and CA, was considered in this study but ultimately abandoned for several reasons. GSH is the most abundant intracellular thiol, with concentrations reaching up to double-digit millimolar values in certain cell types and organs, such as the liver [[38], [39], [40]]. This contrasts sharply with its extracellular concentrations, which are three orders of magnitude lower in plasma [38,40]. High intracellular GSH levels help maintain a reducing environment inside the cell, enabling the export of reduced Hcy. However, low plasma levels of GSH, combined with its low pKa, prevent it from effectively competing for a thiolate bond with Hcy in the generally pro-oxidative environment of plasma [25,38,40]. All studied thiol-based reductants have higher pKa values than GSH, indicating a higher likelihood of successfully competing with Hcy. Additionally, GSH, being a tripeptide, is substantially larger than NAC, MESNA, or CA. This size difference could negatively impact its ability to penetrate deeper into the cavities of plasma proteins to release protein-bound Hcy, compared to the much smaller reductants studied. Lastly, GSH is not an FDA-approved drug, which conflicted with our objective to evaluate only FDA-approved reductants *in vivo*. This approach would facilitate the rapid translation of our results into clinical settings by repurposing current drugs for a novel indication.

Released reduced thiols can alternatively form homo- or hetero-disulfides. It has been hypothesized that a significant amount of plasma Cys is lost in HCU due to the preferred formation of Hcy-Cys heterodisulfides, which are cleared by the kidney [36]. The presence of large quantities of Hcy-Cys heterodisulfide in urine of HCU patients is consistent with this hypothesis [41]. A study using NAC in mild hyperhomocysteinemia showed increased urinary excretion of reduced Hcy as well in addition to the heterodisulfide [35]. Increased cellular export and kidney excretion is, in fact, the principal mechanism of action of CA in the treatment of cystinosis [42] and MESNA has also been shown to have a similar effect [43]. This property of the employed thiol-based reductants could explain why plasma tCys levels did not fully normalize during PEG-CBS co-administration compared to the controls, which did not receive any reductant (Fig. 4C). An alternative view on our data could also indicate that CA may be more specific towards Cys compared to Hcy as plasma tCys levels in CA-treated PEG-CBS-injected cohort of I278T mice were significantly lower compared to the vehicle-, NAC- and MESNA-injected groups. The same line of thinking could also explain why plasma Cth levels after PEG-CBS co-administration were significantly lower in NAC- and MESNA-injected I278T mice compared to the vehicle- and CA-injected cohorts (Fig. 4D). We have excluded the possibility that the reductants would compete with Hcy or Cys as alternative substrates of CBS (Fig. 2G and H). None of the studied thiol-based reductants (NAC, MESNA, CA) were able to replace Ser or Cys as the first CBS substrate forming PLP-aminoacrylate adduct (Fig. 2G) likely due to their physico-chemical properties preventing them to form aminoacrylate intermediate and/or specificity of the catalytic center of CBS. However, CA, but not NAC or MESNA, could serve as an alternative CBS substrate competing with Hcy in a nucleophilic attack of PLP-aminoacrylate adduct likely forming thialysine [44]. This would explain the lack of efficacy of CA in our mouse study (Fig. 4B), making CA less attractive for co-administration with PEG-CBS ERT than NAC or MESNA.

Taken together, the data presented in the current report provide further insight into the mechanism of action of CBS-based ERT and extend our original hypothesis that pegtibatinase acts as a Hcy metabolic sink [10]. Previously, we showed that pegtibatinase can decrease and maintain plasma tHcy levels below a clinically meaningful threshold of 100 μM [8] without any dietary measures or supplementations [12,13,16]. However, normalization of plasma tHcy levels represented an elusive target, which we were unable to achieve even after a substantial increase in the dose of pegtibatinase [16]. Full normalization of plasma tHcy by pegtibatinase was only achieved through a combination with a mild dietary Met restriction [15]. We hypothesized that mild Met restriction (50 % of normal Met content) in a less severe HCU mouse model [15] or severe Met restriction (5 % of normal Met content) in I278T mice [14] lead to a decreased production of Hcy in cells and its export (flux) to plasma consequently yielding substantially lower plasma tHcy concentrations compared to no dietary protein/Met restriction, although the plasma tHcy levels still remained above the 100 μM threshold. More importantly, co-administration of pegtibatinase was able to fully normalize these lower plasma tHcy levels [14,15]. These findings indicated that efficacy of pegtibatinase is not limited by the absolute substrate concentrations, but rather, by the availability of Hcy. Therefore, we conclude that the mechanism of action of CBS-based ERT has three layers. First, upon subcutaneous administration and distribution in plasma, PEG-CBS instantly degrades any free reduced Hcy. Second, cell and tissues export excessively generated Hcy in its reduced form into the bloodstream where it is swiftly catabolized by PEG-CBS. Third, PEG-CBS eliminates Hcy released from the disulfides and from plasma proteins. The second and third layers of this proposed mechanism are affected by other factors, such as metabolic flux of Hcy from tissues or presence of a reducing agent tipping the balance in favor of the reduced Hcy, which in turn represent a substrate for CBS. Therefore, pharmacological alteration of plasma redox dynamics in HCU by biological thiol-based reductants can increase the availability of the reduced Hcy to CBS and thus could potentially increase efficacy of the administered CBS-based ERT, such as pegtibatinase.

## CRediT authorship contribution statement

**Thilo Magnus Philipp:** Writing – review & editing, Writing – original draft, Visualization, Methodology, Investigation, Formal analysis. **Teodoro Bottiglieri:** Writing – review & editing, Methodology, Investigation. **Wilmelenne Clapper:** Writing – review & editing, Resources. **Kai Liu:** Writing – review & editing, Resources. **Steve Rodems:** Writing – review & editing, Resources. **Csaba Szabo:** Writing – review & editing, Resources, Conceptualization. **Tomas Majtan:** Writing – review & editing, Writing – original draft, Visualization, Resources, Project administration, Methodology, Investigation, Funding acquisition, Conceptualization.

## Declaration of competing interest

TM is an inventor on patents related to pegtibatinase, provides ad-hoc consulting to Travere Therapeutics and receives research support from Travere Therapeutics. TB receives compensation for metabolomic analyses from Travere Therapeutics. WC, KL and SR are current or former employees and stockholders of Travere Therapeutics, which clinically develops pegtibatinase as an enzyme replacement therapy for classical homocystinuria. TMP and CS declare no conflicting interests.

## Data Availability

Data will be made available on request.
